# Exploring the Impact of Flavonoids on Symptoms of Depression: A Systematic Review and Meta-Analysis

**DOI:** 10.3390/antiox10111644

**Published:** 2021-10-20

**Authors:** Sawan Ali, Graziamaria Corbi, Michael Maes, Giovanni Scapagnini, Sergio Davinelli

**Affiliations:** 1 Department of Medicine and Health Sciences “V. Tiberio”, University of Molise, 86100 Campobasso, Italy; ali.sawani90@gmail.com (S.A.); graziamaria.corbi@unimol.it (G.C.); giovanni.scapagnini@unimol.it (G.S.); 2 Department of Psychiatry, Faculty of Medicine, Chulalongkorn University, Bangkok 10330, Thailand; dr.michaelmaes@hotmail.com

**Keywords:** flavonoids, depression, antioxidants, oxidative stress, diet, mental health

## Abstract

Recent evidence suggests that diet modifies key biological factors associated with the development of depression. It has been suggested that this could be due to the high flavonoid content commonly found in many plant foods, beverages and dietary supplements. Our aim was to conduct a systematic review to evaluate the effects of dietary flavonoids on the symptoms of depression. A total of 46 studies met the eligibility criteria. Of these, 36 were intervention trials and 10 were observational studies. A meta-analysis of 36 clinical trials involving a total of 2788 participants was performed. The results showed a statistically significant effect of flavonoids on depressive symptoms (mean difference = −1.65; 95% C.I., −2.54, −0.77; *p* < 0.01). Five of the 10 observational studies included in the systematic review reported significant results, suggesting that a higher flavonoid intake may improve symptoms of depression. Further studies are urgently required to elucidate whether causal and mechanistic links exist, along with substantiation of functional brain changes associated with flavonoid consumption.

## 1. Introduction

Depressive disorders are recognized as some of the main causes of disability globally. The World Health Organization (WHO) estimates that 322 million people around the world demonstrate symptoms of depression, nearly 4.4% of the global burden of all diseases [1]. Depressive disorders include two main sub-categories: major depressive disorder (MDD)/depressive episode and dysthymia (i.e., chronic form of mild depression). In both cases, the manifestation of symptoms severely limits psychosocial functioning, diminishes the quality of life and increases the risk of suicidal behaviors [2,3]. Despite intense ongoing research efforts, the exact etiology of depressive disorders remains elusive. However, the complex biological pattern underlying depressive symptoms is thought to be associated with biochemical, genetic and environmental factors, including exposure to physical, social and psychological stressors during early and adult life [4].

The brain appears to be more susceptible than other organs to free radical attacks. Overproduction of free radical species may impair neuronal membrane phospholipids and consequently affect serotonergic and catecholaminergic receptor functions [5]. An altered redox status has been confirmed in the central nervous system (CNS) of depressed patients and oxidative disturbances have been associated with lowered concentrations of several endogenous antioxidant enzymes [6]. Pharmacological treatments, although proven to be effective in treating from moderate to severe symptoms in MDD, have only modest effect sizes and approximately 30% of depressed patients do not respond sufficiently to the existing drug therapies. Moreover, antidepressants are associated with a plethora of side effects and a propensity for abuse [7].

Nutritional psychiatry is an emerging discipline that focuses on the role of diet and nutrients in diverse aspects of mental health [8]. Numerous mechanisms were identified through which diet and its components may affect depression. These include the modulation of the pathways involved in inflammation, oxidative stress, epigenetics, mitochondrial dysfunction, the gut microbiota, tryptophan–kynurenine metabolism, the hypothalamic–pituitary–adrenal (HPA) axis and neurogenesis [9]. Although Molendijk et al. highlighted various issues regarding study recruitment and blinding, the SMILES trial showed that a 12-week healthy diet intervention improved ratings of depression in adults with a mean age of 40 years [10,11]. Further randomized clinical trials (RCTs) confirmed the benefits of a Mediterranean-style diet on mental health in depression, namely the HELFIMED and PREDIMED trials [12,13]. A meta-analysis of 16 RCTs that involved some type of diet improvement showed that diet interventions overall reduced depressive symptoms [14]. Currently, there are also human studies showing that the antioxidant/anti-inflammatory effects of dietary components may improve cognitive and depression outcomes [15,16].

Flavonoids belong to a family of polyphenolic plant compounds widely present in fruits, vegetables and certain beverages. Based on their molecular structure, flavonoids are classified into six main subclasses that include flavones, flavonols, flavanones, flavan-3-ols, anthocyanins and isoflavones [17]. Experimental studies have repeatedly demonstrated that dietary flavonoids and their representative subclasses exert modulatory effects on key cellular mechanisms associated with endogenous antioxidant systems [18], inhibition of immune-inflammatory pathways [19], antidepressant-like effects and enhancement of mitochondrial functions and neurotransmission [20,21]. Another advantage is that some flavonoids may have some clinical efficacy in individuals with depressive disorders without causing the side effects usually associated with conventional antidepressants [22]. However, to date, there are no meta-analyses synthesizing the relationship between dietary flavonoids and depressive disorders. Hence, the aim of the current paper is to conduct a systematic review and meta-analysis of human studies to critically evaluate whether flavonoids have some clinical efficacy in patients with depressive symptoms.

## 2. Methods

The search strategy, screening and selection criteria were developed according to the Preferred Reporting Items for Systematic Reviews and Meta-Analysis (PRISMA) Statement [23]. The registration number in PROSPERO is CRD42020173649.

### 2.1. Eligibility Criteria and Search Strategies

A literature search was conducted in the following databases: Scopus, PubMed and Web of Science. Observational and clinical studies, which clearly assessed the effect of flavonoids on the symptoms of depression, published up to 30 July 2021, were included in the review. We included articles that determined the flavonoid content of foods or the dose of the flavonoid-containing supplements. Articles were excluded from the review for the following reasons: studies not published in English; articles that used secondary data, such as reviews, meta-analysis, conference papers and book chapters; studies on animal models or in vitro experiments; studies that did not use a depression rating scale. In order to conduct a comprehensive systematic literature search, we used both controlled vocabulary and free text terms. The search was conducted using Boolean operators “AND” and “OR” to combine the following terms: “flavonoid” OR “anthocyanin” OR “anthocyanidin” OR “cyanidin” OR “delphinidin” OR “malvidin” OR “pelargonidin” OR “peonidin” OR “petunidin” OR “flavan-3-ol” OR “catechin” OR “epicatechin” OR “epigallocatechin” OR “gallocatechin” OR “proanthocyanidin” OR “theaflavin” OR “thearubigin” OR “flavonol” OR “isorhamnetin” OR “kaempferol” OR “myricetin” OR “quercetin” OR “flavone” OR “apigenin” OR “luteolin” OR “baicalein” OR “chrysin” OR “flavanone” OR “eriodictyol” OR “hesperetin” OR “naringenin” OR “isoflavone” OR “daidzein” OR “genistein” OR “glycitein” OR “biochanin” OR “formononetin” AND “depression” OR “depressive disorder” OR “depressive symptom” OR “antidepressant” OR “antidepressive”. At the same time, similar queries were respectively used for controlled vocabulary search: “flavonoids” [Mesh] AND “depression” [Mesh], INDEXTERMS “flavonoids” AND “depression”.

### 2.2. Study Selection and Data Extraction

Two authors (S.D. and S.A.) independently reviewed the titles and abstracts obtained from all the databases. After the removal of duplicate records with a reference management software (EndNote X8; Clarivate Analytics, Philadelphia, PA, USA) the titles and abstracts were reviewed. The full texts were screened by two authors (S.D. and G.C.) and all articles that did not meet the inclusion criteria were excluded. In the case of disagreement about the eligibility of a study, a third author (G.S.) decided which articles were included. Data were extracted independently by two authors (S.D. and S.A.) and confirmed where necessary by the principal investigator (G.S.). A data extraction spreadsheet was developed and the information from the studies included in the review was extracted and tabulated. The following information was recorded: author’s name, publication year, study country, study design, participant characteristics (sample size, gender and age), health status, duration of follow-up, outcome assessment for depression, intervention (type of compounds and dose) and results.

### 2.3. Study Quality Assessment

The study quality and risk of bias of each included study were assessed using the Cochrane risk of bias tool for RCTs [24] and the Newcastle–Ottawa Scale (NOS) for observational studies [25]. The Cochrane risk of bias tool is made up of 7 components: (1) sequence generation, (2) allocation sequence concealment, (3) blinding of participants and personnel, (4) blinding of outcome assessment, (5) incomplete outcome data and (6) selective outcome reporting. The NOS, which was identified as one of the most useful tools for assessing methodological quality of observational studies, evaluates three quality domains (selection, comparability and outcome) divided across eight specific items. The NOS assigns a maximum score of 9 points. Studies with NOS scores of 0–3, 4–6 and 7–9 were considered low, moderate and high quality, respectively.

### 2.4. Statistical Analysis

The meta-analysis was carried out using the R Software, version 4.0.3 (R Foundation for Statistical Computing, Vienna, Austria) and the interface R-Studio version 1.4.1717 (R studio, PBC, Boston, MA, USA). A *p* value < 0.05 was considered to be statistically significant. Continuous data from the studies included were expressed as mean difference with a 95% confidence interval. Participants who consumed the flavonoid intervention were recorded as the experimental group, while those who consumed the placebo intervention were recorded as the control group. Since measurement time periods differed among studies, final measurement time points of each study were used. The summary statistics required for each outcome were the number of participants in the intervention and control groups at baseline and post-test, the mean change from baseline and the SD of the mean change. If change-from-baseline scores were not provided, they were calculated using baseline and post-test means and SDs. The mean difference was used to express the results across studies. Statistical heterogeneity was calculated using the Higgins I^2^ statistic, which describes the percentage of variability in the effect estimate due to heterogeneity rather than sampling error. Inconsistency was examined using I^2^ and the following grades were applied: <25% (very low), from 25 to <50% (low), from 50 to <75% (moderate) and ≥75% (large) [26]. A random effects model was chosen for the meta-analyses because this method of analysis is favored when there is evidence of heterogeneity among studies. In order to assess whether the pooled estimate was biased by the effect of any particular study, we also carried out a sensitivity analysis by removing outlier studies and recalculating the pooled estimate. Subgroup analyses were also performed to evaluate the potential effect of different factors on the depressive outcomes. These factors were flavonoid subclasses, type of clinical trials (controlled and uncontrolled) and level of blinding. The visual inspection of funnel plots and Egger’s linear regression were used to assess the publication bias [27,28].

## 3. Results

### 3.1. Study Selection

**Figure 1:**
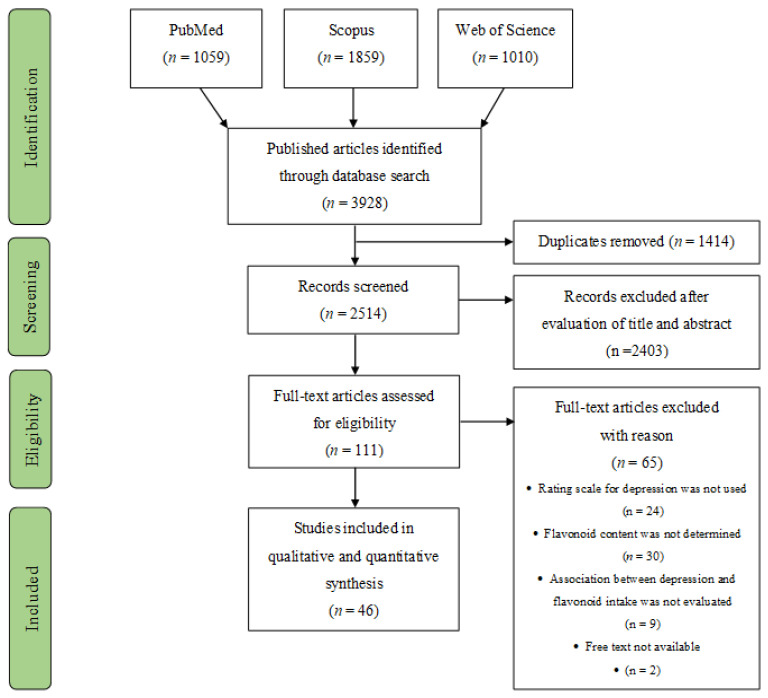
PRISMA flow diagram for systematic review and meta-analysis.

As presented in Figure 1, the combined search resulted in 3928 published articles from the three databases, among which 1414 were duplicates. During the screening phase, 2514 records were discarded because they clearly did not meet the inclusion criteria, as determined from the title and abstract. The remaining 111 articles were examined for eligibility assessment through full-text reading. Of these, 65 records did not meet the eligibility criteria, or the full text was unavailable. Thus, a total of 46 studies were included in the final analyses.

### 3.2. Study Characteristics

The 46 included studies were conducted between 1999 and 2021. Thirty-six out of the 46 studies selected were clinical trials [29,30,31,32,33,34,35,36,37,38,39,40,41,42,43,44,45,46,47,48,49,50,51,52,53,54,55,56,57,58,59,60,61,62,63,64] and 10 studies were observational cohort studies [65,66,67,68,69,70,71,72,73,74]. Among the 36 included clinical trials, 31 were RCTs with an average number of 67.4 randomized participants. The clinical trials varied in time duration from as few as 5 days to 2 years with the most common time frame being 8 weeks. The observational studies included both longitudinal cohort and cross-sectional designs and had a mean of 9426 participants. Most of the studies assessed both genders (*n* = 25), 20 assessed only females and only 1 study assessed only men. The age of all participants varied widely from 18 to 92 years. However, the majority of studies included subjects aged ≥40 years.

Most of the studies measured depression symptomatology in disease states, including osteopenia [52], osteoarthritis [45], fibromyalgia [55], multiple sclerosis [47,63], neurodegeneration or age-associated cognitive decline [32,38,39,54], obesity/overweight [34,49], schizophrenia and bipolar disorder [43,56] and chronic hepatitis C infection [44]. Sixteen of the studies were in perimenopausal, menopausal, or postmenopausal women [31,33,35,36,37,42,48,50,51,52,53,61,62,64,70,71]. A summary of the characteristics of all the included studies are presented in Table 1 and Table 2.

### 3.3. Flavonoids

A wide variety of flavonoids was assessed in the research reports included in this review. The majority of clinical studies assessed the effect of flavonoids consumed via tablets or dry extract capsules [29,30,31,32,33,34,35,36,39,40,41,42,43,44,45,47,48,49,50,51,52,53,54,56,57,58,59,60,61,62,64], liquids [38,46,55,63] and powder [37]. The observational studies looked at flavonoids consumed in their biological whole food form. The exposure was measured using the diet history questionnaire (DHQ) [65,68,69,74] and food frequency questionnaire (FFQ) [66,67,70,72,73]. Only 1 observational study measured urinary concentrations of isoflavones using liquid chromatography coupled with tandem mass spectrometry [71]. The most commonly tested groups of flavonoids in the intervention trials were isoflavones [31,32,33,35,36,37,42,48,50,51,52,55,61,62,64]. Although several clinical studies assessed total flavonoid intake or considered the combined effect of mixed flavonoid subclasses, other classes of flavonoids tested included flavan-3-ols, flavonols, flavones, anthocyanins and proanthocyanidins. Two of the observational studies estimated habitual dietary consumption of total polyphenols and then determined total flavonoid intake [67,72]. In addition to the intake of total flavonoids, two observational studies also estimated flavonoid subclasses, such as flavonols, flavones, flavanones, anthocyanins, flavan-3-ols and proanthocyanidins [66,67]. However, the main measures of exposure in the observational studies were isoflavones [65,68,69,70,71,73,74].

### 3.4. Depression Scales

As shown in Table 1 and Table 2, a large number of depression scales has been used in the studies included in this review. The most common depression scales used in both clinical and observational studies were the Geriatric Depression Scale (GDS), Center for Epidemiological Studies Depression Scale (CES-D), Beck Depression Inventory (BDI) and Hamilton Depression Scale (HAM-D). The GDS was used in 6 clinical trials [29,32,37,38,50,54] and 3 observational studies [65,66,73] of the 46 included studies. The GDS is a commonly used depression scale in geriatric populations. This scale has several versions and it can be applied to normal, medically ill or cognitively impaired older adults [75]. Another popular depression scale used was the CES-D, which was used in three clinical trials [46,55,64] and five observational studies [66,67,68,69,70]. The CES-D, designed for large-scale studies, is a 20-item self-report inventory that can be completed in approximately 5 min [76]. The BDI was used in seven intervention trials [34,44,45,47,48,49,61]. The BDI is a 21-item, multiple-choice inventory designed to assess the level of key depressive symptoms (e.g., guilt, low self-worth and suicidal ideation) in adults [77]. Another popular scale was the HAM-D, which was used in five of the studies [30,31,43,56,62]. This scale consists of 17 items that are rated by the observer, rather than the patient [78].

### 3.5. Flavonoids and Depressive Symptoms

Overall, dietary flavonoids were associated with an improvement in depressive symptoms. The majority of trials (*n* = 22) found significant effects of consuming flavonoids on the symptoms of depression [29,30,31,33,34,35,36,41,42,44,46,47,48,49,51,52,56,58,60,61,62,64]. Zarghami et al. observed a significant decrease in the depression score after flavonoid intervention but the antidepressant drug fluoxetine had a greater anti-depressant activity than flavonoids [57]. Thirteen clinical studies showed no significant effect after the intervention with flavonoids [32,37,38,39,40,43,45,50,53,54,55,59,63]. Significant results on depressive symptoms associated with perimenopause, menopause, or postmenopause were reported in 11 trials of the 14 studies investigating the effect of flavonoids during the menopause transition [31,33,35,36,42,48,51,52,61,62,64]. One clinical study also noted a statistically significant effect on depressive symptoms in postmenopausal women with osteopenia [52]. Two out of the three trials conducted on anxious individuals found a significant improvement in depressive symptoms after treatment with flavonoids [30,41]. There were also significant effects of flavonoids on overweight or obese adults [34,49] and subjects affected by multiple sclerosis [47], chronic hepatitis C infection [44] and bipolar disorder [56]. No effects were reported in Alzheimer’s disease or individuals affected by age-associated memory impairment. Five out of the 10 observational studies reported that higher flavonoid intakes are associated with lower depression symptoms [66,67,68,69,74]. No significant associations were found between flavonoid intake and depressive symptoms in elderly subjects, menopausal women and subjects affected by mental health problems.

### 3.6. Meta-Analysis of the Effect of Flavonoids on Depressive Symptoms

**Figure 2:**
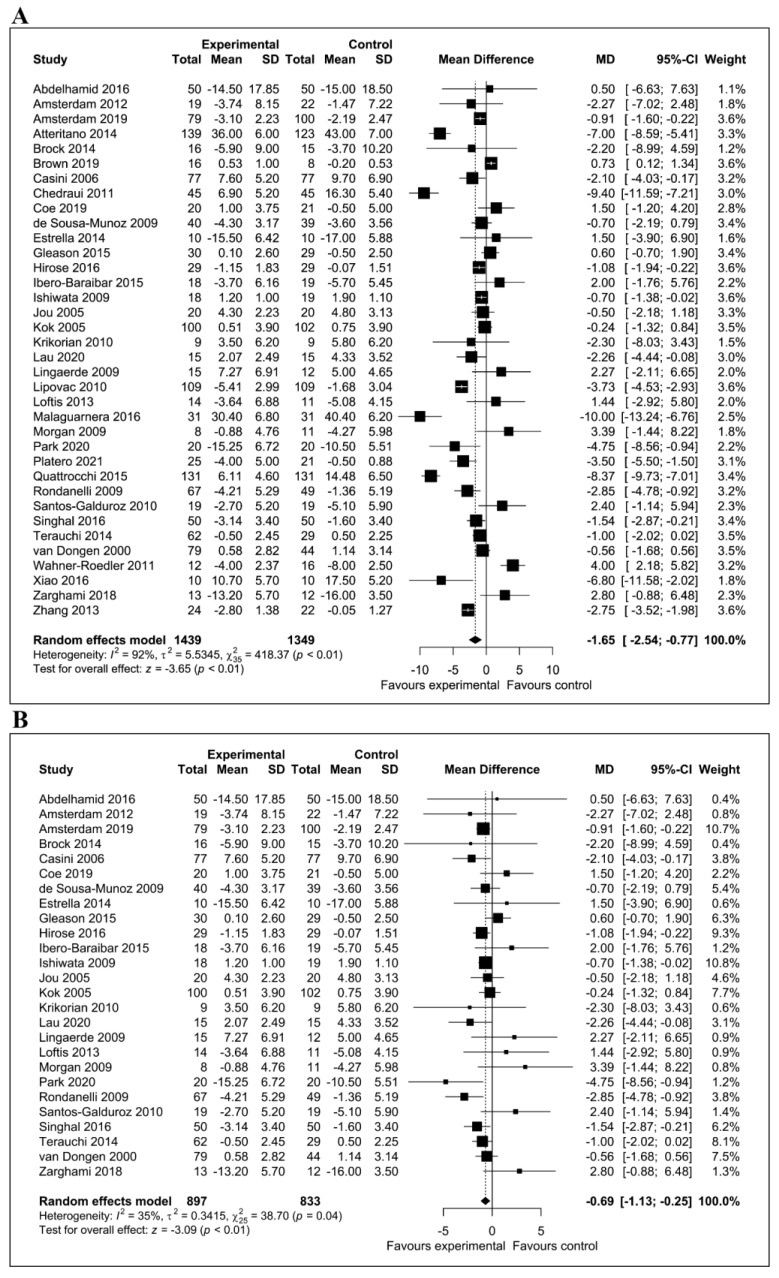
Forest plots showing the effects of flavonoid consumption on depressive symptoms. (**A**) Forest plot of the effect of dietary flavonoids on depressive symptoms in 36 clinical trials. (**B**) Sensitivity analysis summarizing the effect of flavonoids on depressive symptoms.

**Figure 3:**
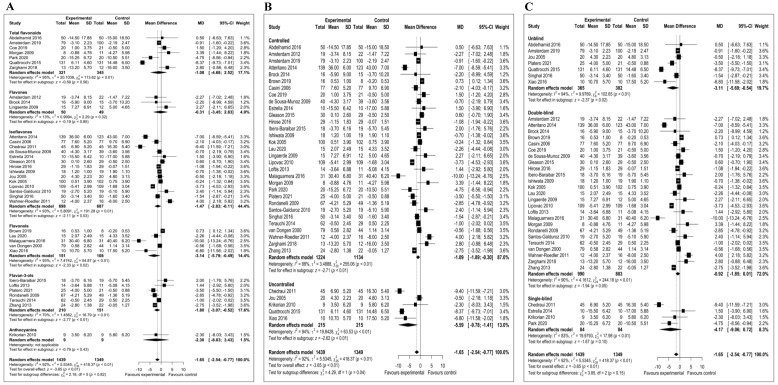
Forest plots for subgroup analyses. (**A**) Forest plot based on the type of flavonoid subclass; (**B**) type of clinical trial (controlled and uncontrolled) and (**C**) level of blinding.

Overall, we meta-analyzed 36 trials involving a total of 2788 participants. Observational studies were not included in the meta-analysis because sufficient data were not available. The meta-analysis showed a statistically significant reduction of depressive symptoms after consumption of dietary flavonoids (mean difference = −1.65; 95% CI, −2.54, −0.77; *p* < 0.01). Figure 2A depicts a forest plot of the effect of flavonoids on the reduction in depressive symptoms compared with controls. There was evidence of significant heterogeneity among studies (I^2^ = 92%). We carried out a sensitivity analysis by omitting outlier studies and recalculating the effect size. Using a random effect model, the sensitivity analysis did not affect the overall findings. By eliminating 10 of the studies from the primary analysis, the positive effects of flavonoids on depression symptoms remained significant (mean difference = −0.69; 95% CI, −1.13, −0.25; *p* < 0.01) (Figure 2B). After excluding outlier studies, the heterogeneity was reduced to I^2^ = 35%. The subgroup analyses were conducted based on the type of flavonoid subclass, type of clinical trial (controlled and uncontrolled trials) and level of blinding. As presented in Figure 3A, isoflavone (mean difference = −1.47; 95% CI; −2.83, −0.11; *p* = 0.03), flavonols (mean difference = −3.14; 95% CI, −5.79, −0.49; *p* = 0.02) and flavan-3-ol (mean difference = −1.80; 95% CI, −3.07, −0.52; *p* < 0.01) (Figure 3A) subclasses showed statistically significant effects. In the subgroup analysis based on the presence or absence of control group, pooled results from 31 controlled studies involving 2358 participants were significant (mean difference = −1.09; 95% CI, −1.89, −0.30; *p* < 0.01). Likewise, the effect size of five studies conducted with no control was also significant (mean difference = −5.59; 95% CI, −9.78, −1.41; *p* < 0.01) (Figure 3B). The subgroup analysis based on the level of blinding revealed a significant effect of flavonoids in 25 double-blind studies (mean difference = −0.92; 95% CI, −1.85, −0.01; *p* = 0.05) and 7 unblind intervention trials (mean difference = −3.11; 95% CI, −5.69, −0.54; *p* = 0.02). (Figure 3C).

### 3.7. Publication Bias

**Figure 4:**
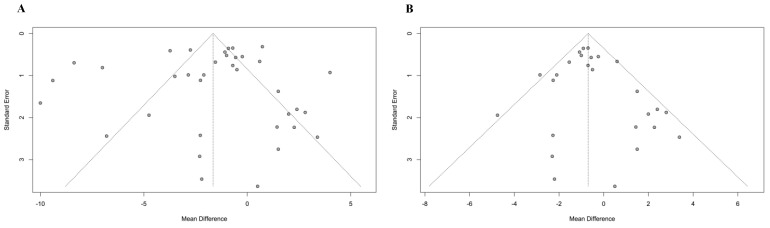
Funnel plots for publication bias. (**A**) Primary analysis with 36 studies and (**B**) sensitivity analysis with 26 studies.

The publication bias was assessed using funnel plots and Egger’s test. The visual inspection of the funnel plot revealed a symmetrical distribution of studies (Figure 4A,B). Additionally, Egger’s test (*p* = 0.54) did not show significant publication bias. Thus, the likelihood of publication bias was low. Results for the risk of bias are shown in Supplementary Figure S1 and Supplementary Table S1. Overall, results from the critical appraisal tool show good methodology in the intervention trials. Most included clinical studies (25 of 36) were double-blind. However, although these trials claimed to be double blinded, many failed to provide details of how the investigators and those delivering the interventions were blinded in the method sections. The majority of trials were randomized, but most of the studies did not indicate the technique for sequence generation. A common weakness observed in the clinical trials was also the lack of information regarding the methods for evaluating or verifying participant compliance. Almost all manuscripts reported the number of dropouts during the interventions. The study qualities of the selected observational studies were diverse. Based on the NOS quality assessment results, eight studies achieved NOS scores between 4 and 6 (moderate quality) [65,67,68,69,70,71,72,73], one study was rated as “low” quality (3 points) [74] and one study was rated as “high” quality (8 points) [66]. This variation in quality of observational studies could possibly be explained by the following reasons: (a) no selection of the unexposed cohort; (b) no reliability of outcome assessment; (c) 20% or over losses to follow-up after baseline.

## 4. Discussion

A recent study highlighted the importance of a systematic method for summarizing the evidence on dietary interventions in depression [79]. Currently, systematic reviews and meta-analyses have shown that unhealthy dietary patterns are associated with an increased risk of depression, while high-quality diets are related to a lower risk of depressive symptoms [15,80]. In addition, several studies have indicated that nutritional deficiencies, such as vitamins (B vitamins), minerals (magnesium, selenium, zinc and iron) and amino acids, are common in depressed patients [81,82,83]. Although hotly debated, recent findings have also suggest that omega-3 polyunsaturated fatty acids, vitamin D and minerals may have a protective effect against the development of depressive symptoms [84,85,86].

Dietary flavonoids possess a large number of neuroprotective actions in various pathophysiological conditions, including depression. Although the antidepressant effects of flavonoids have been confirmed in various animal models of depression [22,87], the results of human studies remain controversial. The purpose of this manuscript is to provide a comprehensive review of the literature regarding the effects of flavonoids on depressive symptoms. A considerable number of the included studies observed significant results, suggesting that dietary flavonoids may improve depressive symptoms.

Our meta-analysis pooled results from 36 clinical trials to estimate the effects of the consumption of flavonoids on outcomes associated with depressive symptoms. The results show a significant antidepressant effect of flavonoids on subjects affected by depressive symptoms. However, the 36 clinical trials included in our meta-analysis were associated with a considerable heterogeneity. The sensitivity analysis restricted to 26 clinical studies also found a significant improvement in depressive symptoms with a low level of heterogeneity.

In the subgroup analyses by flavonoid subclasses, we only found a significant effect of isoflavones, flavonols and flavan3-ols on depressive symptoms. Likewise, separate subgroup analyses confined to double-blind and unblind trials showed a significant effect of flavonoids against depressive symptoms. Additional subgroup analyses of controlled and uncontrolled clinical studies also revealed a statistically significant effect of flavonoids.

Most of the trials examined here used different treatment durations and involved a large variety of dosages and sources of flavonoids. Depressive symptoms were frequently accompanied by other conditions and, in many cases, the effect of flavonoids on depressive symptoms was not always the primary outcome of the study. Moreover, many different types of depression scales were used. There were no large-scale randomized trials and most of the intervention studies were underpowered. It should be also noticed that the capture of the dietary background through an initial dietary assessment was not performed in the included clinical studies. This is crucial to evaluate flavonoid content in the habitual diet (i.e., listing the food sources of flavonoids), avoid underestimation of flavonoid exposure and know whether the planned flavonoid-based intervention contains a flavonoid intake level sufficiently above the expected background intake. Indeed, when there was evidence of effectiveness of flavonoids, it was not clear that the flavonoids themselves (rather than other bioactive components consumed as part of the diet) are solely or partially responsible for the observed effects. Therefore, the results from these clinical studies do not provide a clear picture on optimal dosage, treatment duration and estimated flavonoid intakes. However, although our findings should be interpreted cautiously, this meta-analysis provides preliminary evidence that treatments with flavonoids may potentially have some clinical efficacy in the treatment of depressive symptoms.

The observational studies reported the flavonoid intake of individuals in a real-life setting and estimated the prevalence of depressive symptoms among consumers of flavonoids. Although observational studies were not included in the meta-analyses as they did not present statistically homogeneous categories, five studies found significant results [66,67,68,69,74], suggesting that a higher flavonoid intake may improve symptoms of depression. Flavonoid intake was measured with different FFQ and DHQ. However, there are some issues with these tools, such as under- or over-reporting consumption and measurement error [88]. Additionally, errors of exposure measurement may be due in large part to the lack of analytic values for specific flavonoid classes. Except for one study [71], none of these observational studies took advantage of the more recent flavonoid analytic techniques and biomarkers of intake were not used. Moreover, most of the studies focused on intake of a specific flavonoid-rich food (typically, soy) or adherence to dietary patterns [65,68,69,73,74] and they did not perform a comprehensive evaluation of flavonoid subclasses. Therefore, all these factors must be considered when interpreting the results.

Both clinical trials and observational studies measured depressive symptoms rather than MDD and did not fully assess the severity of depressive symptoms. Therefore, the results obtained here cannot be generalized to treatment of MDD. Although the specific links between flavonoids and depression are not fully clarified, several mechanisms have been suggested [89]. Extensive evidence indicates that depression is associated with activated immune-inflammatory, neuro-oxidative and neuro-nitrosative pathways [90]. Animal studies have demonstrated that flavonoids possess an antidepressant-like property via interactions with oxidative pathways and antioxidant systems [21]. Flavonoids may also exert beneficial effects on depression through their anti-oxidants and anti-inflammatory properties and inhibition of proinflammatory mediators [91,92,93]. Another possible mechanism is that flavonoids may modulate signaling pathways, which are responsible for maintaining neuron survival and synaptic plasticity [94,95,96]. It is important to note that the beneficial effects of flavonoids may depend on their bioavailability, which differs greatly by subclasses. Bioavailability is influenced by absorption, metabolism and disposition in tissues and cells. The structure of flavonoids influences the rate and extent of intestinal absorption which, in turn, affects the metabolites circulating in the plasma. Flavonoids are metabolized by colonic microflora and several studies suggest that the metabolites of flavonoids may be one of the characteristics responsible for their therapeutic effects [97]. The differences in bioavailability and absorption rates of various flavonoids are lacking in the included studies and this aspect should be considered when interpreting the results. Moreover, flavonoid content between foods and supplements is highly variable and the type of source (i.e., foods or supplements) may affect numerous factors associated with bioavailability and health outcomes [98,99]. A common theme in this review is the use of isoflavones for peri- and postmenopausal women and during pregnancy. Isoflavones are found mainly in soy-based foods and they exhibit estrogenic activity by binding estrogen receptors. It has been suggested that isoflavones may alleviate the symptoms of depression by acting as phytoestrogens; therefore, they may only be appropriate for use in specific population groups [100,101].

Our manuscript has several strengths. First, to our knowledge, this is the first paper which systematically reviewed available human studies on the effects of flavonoids on depressive symptoms. The literature search was conducted applying specific inclusion and exclusion criteria and using controlled vocabulary queries to identify relevant studies. The publication bias was evaluated with the visual inspection of the funnel plot and Egger’s test. The visual inspection of the funnel plot of studies included in the sensitivity analysis showed no evidence of publication bias. Furthermore, a quality assessment of the observational studies and clinical trials was performed and most of the included studies had a relatively good quality.

Recent research efforts are beginning to highlight differences in which men and women respond to diet and express symptoms of depression. The interactions between flavonoid intake, gender and depressive symptoms was not tested in any of the studies. No clinical study investigated the association between flavonoid consumption and neurochemical factors, which are substantial contributors to the pathogenesis of depression. Moreover, social and cultural factors were not considered as contributory factors to the development of depressive symptoms.

## 5. Conclusions

Based on our findings, we may provide some directions for future research delineating the effects of flavonoids on depressive phenomenology and MDD. First, given that flavonoids and their subclasses are commonly consumed as part of a normal diet, more carefully designed studies should be performed to improve dietary intake assessment and determine the specific effects on symptoms of depression. Second, well-powered and long-term trials are required to explore the optimal dosage and treatment duration. Third, studies investigating biomarkers of intake are also required to clarify interactions of flavonoids with potentially causal mechanisms. Fourth, prospective studies are needed to confirm the association between flavonoid consumption and depressive symptoms or MDD and between immune and nitro-oxidative biomarkers of depression in order to decipher whether the clinical efficacy of flavonoids is associated with an improvement of those biomarkers. If confirmed, the findings may have important implications for depression prevention.

## Figures and Tables

**Table 1:** Data summary of clinical intervention trials assessing the effects of flavonoids on depressive symptoms.

Study (Author, Year, Country, Ref.)	Study Design	Subjects	Condition	Intervention	Depression Scale	Results
Abdelhamid et al., 2016; Egypt [29]	Randomized clinical trial Duration: 24 weeks	100 subjects (60–70 y) (*n* = 50 women; *n* = 50 men)	Mild depression	Flavonoid-rich extract from date palm fruit (rutin 11.2 mg/100 mg)	GDS	Significant effect compared with baseline (*p* = 0.0001)
Amsterdam et al., 2012; US [30]	Randomized double-blind placebo-controlled trial Duration: 8 weeks	41 subjects (mean age 42.9 y) (*n* = 26 women; *n* = 15 men)	Anxiety with or without depression	Chamomile capsules (220 mg/d; 1.2% apigenin)	HAM-D	Significant effect (treatment vs. placebo *p* < 0.05)
Amsterdam et al., 2019; US [41]	Randomized open-label trial Duration: 8 weeks	179 subjects (mean age 45.7 y) (*n* = 119 women; *n* = 60 men)	Anxiety with or without depression	Chamomile capsules (1500 mg/d; 18 mg of flavonoids)	HRSD	Significant effect in subjects with depression (*p* < 0.02)
Atteritano et al., 2014; Italy [52]	Randomized double-blind controlled trial Duration: 2 years	262 women (49–67 y)	Osteopenia and Postmenopause	Genistein tablets (54 mg/d)	ZSDS	Significant effect (treatment vs. placebo *p* < 0.01)
Brock et al., 2014; UK [59]	Randomized double-blind placebo-controlled crossover trial Duration: 2 weeks	31 subjects (mean age 34 y) (*n* = 25 women; *n* = 6 men)	Anxious and non-anxious population	*Scutellaria lateriflora* capsules (350 mg/d; 11.7 mg/g baicalein; 7.7 mg/g wogonin)	POMS	No significant effect
Brown et al., 2019; US [60]	Randomized double-blind placebo-controlled trial Duration: 5 days	24 subjects (18–50 y) (men and women)	Healthy subjects	Icariin capsules (from 100 to 1.680 mg/d)	QIDS-C	Significant effect (treatment vs. placebo *p* = 0.02)
Casini et al., 2006; Italy [61]	Randomized double-blind crossover placebo-controlled study Duration: 6 months	77 women (49–50 y)	Postmenopause	Phytoestrogens tablets (soy isoflavones 60 mg/d; 45% genisytein 45% daidzein 10% glycitein)	BDI and POMS	Significant effect in BDI and POMS scores (treatment vs. placebo BDI, *p* < 0.01; POMS, *p* < 0.001)
Chedraui et al., 2011; Ecuador [62]	Single blinded pilot clinical trial Duration: 3 months	45 women (age 40–59 y)	Menopausal symptoms	Soy isoflavone capsules (100 mg/d; 40% isoflavones)	HAM-D	Significant effect compared with baseline (*p* < 0.05)
Coe et al., 2019; UK [63]	Randomized, double-blind placebo- controlled trial Duration: 6 weeks	41 subjects (mean age 44 y) (*n* = 30 women; *n* = 10 men)	Relapsing and remitting multiple sclerosis	Flavonoid-rich cocoa drink (intervention group 200 mg/day)	HADS	No significant effect
de Sousa-Muñoz, and Filizola, 2009; Brazil [64]	Placebo-controlled double-blind randomized trial Duration:16 weeks	79 women (mean age 53.3 y)	Depressive symptoms in climacteric syndrome	Soy isoflavone capsules (120 mg/d; 60 mg isoflavones; 20 mg daidzein; 14 mg genistein)	CES-D	Significant effect compared with baseline (*p* = 0.001)
Estrella et al., 2014; Chile [31]	Randomized clinical trial Duration: 3 months	20 women (45–55 y)	Depression in menopause	Soybean concentrate (100 mg/d; 50 mg isoflavones)	HAM-D and ZSDS	Significant effect in depression scores compared with baseline (*p* < 0.001)
Gleason et al., 2015; US [32]	Randomized double-blind parallel group clinical trial Duration:6 months	59 subjects (>60 y) (men and women)	Alzheimer’s disease	Soy isoflavone capsules (100 mg/d; 85% daidzein, and genistein)	GDS and POMS	No significant effect
Hirose et al., 2016; Japan [33]	Randomized double-blind placebo-controlled trial Duration 8 weeks	58 women (40–60 y)	Menopause	Soy isoflavone tablets (12.5 mg/d; 25 mg/d; genistein 51.8%, daidzein 43.3%, glycitein 4.9%)	HADS	Significant effect in compared with placebo (*p* = 0.033)
Ibero-Baraibar et al., 2015; Spain [34]	Randomized double-blind placebo-controlled parallel trial Duration: 4 weeks	37 subjects (mean age 57 y) (*n* = 25 women; *n* = 22 men)	Overweight or obese adults	Cocoa extract (1.4 g/d; 414 mg flavan-3-ols; 153 mg epicatechin; 15 mg catechin; 246 mg procyanidins),	BDI	Significant effect compared with baseline (*p* < 0.01)
Ishiwata et al., 2009; Japan [35]	Randomized double-blind placebo-controlled trial Duration: 12 weeks	37 women (mean age 46.6 y)	Menopause	Equol capsules (10 or 30 mg/d)	POMS	Significant effect compared with placebo (*p* < 0.05)
Jou et al., 2005; Taiwan [36]	Single-centre prospective randomized trial Duration: 6 weeks	20 women (age N/A)	Menopause	Soy isoflavone capsules (35 or 70 mg/d isoflavones)	GCS	Significant effect in 35 mg groups compared with baseline (*p* < 0.05)
Kok et al., 2005; Netherlands [37]	Randomized double-blind placebo-controlled trial Duration: 12 months	202 women (60–75 y)	Postmenopause	Soy powder (25.6 g/d; 52 mg/g genistein; 41 mg/g daidzein; 6 mg/g glycitein)	GDS	No significant effect
Krikorian et al., 2010; Canada [38]	Single-blind placebo-controlled trial Duration:12 weeks	9 subjects (mean age 76.2) (men and women)	Memory decline	Blueberry juice (three doses; 444 mL/d, 0.42 g anthocyanins; 532 mL/d, 0.51 g anthocyanins; 621 mL/d, 0.59 g anthocyanins)	GDS	No significant effect
Lau et al., 2020; Malaysia [39]	Multi-center randomized double-blind placebo-controlled trial Duration: 6 months	30 subjects (mean age 66.4 y) (*n* = 23 women; *n* = 7 men)	Mild cognitive impairment	*Persicaria minor* extract (500 mg/d; 0.45% quercetin-3-glucuronide; 0.15% quercitrin)	POMS	No significant effect
Lingaerde et al., 1999; Norway [40]	Randomized double-blind placebo-controlled trial Duration: 10 weeks	27 subjects (26–58 y) (*n* = 21 women; *n* = 6 men)	Seasonal affective disorder	*Ginkgo biloba* tablets (120–160 mg/d; 24 mg flavones)	MADRS	No significant effect
Lipovac et al., 2010; Austria [42]	Randomized double-blind placebo-controlled trial Duration: 3 months	109 women (mean age 53.5 y)	Postmenopausal symptoms	Isoflavone capsules (80 mg/d; extract in form of	HADS and ZSDS	Significant effect in HADS and ZSDS scores compared
Loftis et al., 2013; US [43]	Randomized double-blind placebo-controlled trial Duration: 8 weeks	25 subjects (≥18 years) (men and women)	Schizophrenia and bipolar disorder	biochanin A, formononetin, genistein and daidzein) EGCG capsules (N/A mg/d; capsules enriched with 150 mg of theaflavin)	HAM-D	with control (*p* < 0.001) No significant effect
Malaguarnera et al.; 2016; Italy [44]	Randomized double-blind placebo-controlled trial Duration: 1 year	62 subjects (mean age 46 y) (*n* = 26 women; *n* = 36 men)	Hepatitis C virus infection	Silybin pills (active group: 1.5 mg/kg/week Peg–IFN plus RBV + silybin 94 mg + vitamin E 30 mg + phospholipids 194 mg)	BDI	Significant effect in intervention group compared with placebo (*p* < 0.05)
Morgan et al., 2009; US [45]	Randomized double-blind placebo-controlled trial Duration: 12 weeks	19 subjects (40–75 y) (men and women)	Moderate osteoarthritis	*Scutellaria baicalensis* and *Acacia catechu* pills (500 mg/d; N/A mg/g of flavonoids)	BDI	No significant effect
Park et al., 2020; South Korea [46]	Randomized single-blind controlled trial Duration: 8 weeks	40 subjects (mean age 21.8 y) (*n* = 24 women; n = 16 men)	Depressed and non-depressed population	Flavonoid-rich orange juice (380 mL/d; 600 mg flavonoids)	CES-D	Significant effect compared with baseline (*p* < 0.0001)
Platero et al., 2021; Spain [47]	Randomized placebo-controlled trial Duration: 4 months	46 subjects (mean age 47.1 y) (*n* = 32 women; *n* = 14 men)	Multiple sclerosis	EGCG capsules (800 mg/d) with coconut oil (60 mL/d)	BDI	Significant effect compared with baseline (*p* = 0.007)
Quattrocchi et al., 2015; Italy [48]	Intervention trial Duration: 6 months	131 women (42–67 y)	Menopausal symptoms	Phyto complex (200 mg/d; 20% isoflavones, 1.5% vitexin)	BDI	Significant effect compared with baseline (*p* < 0.05)
Rondanelli et al., 2009; Italy [49]	Randomized double-blind placebo-controlled parallel trial Duration: 2 months	116 subjects (18–50 y) (*n* = 89 women; *n* = 27 men)	Overweight	EGCG capsules (50 mg/d)	BDI	Significant effect compared with placebo (*p* < 0·005)
Santos- Galduróz et al., 2010; Brazil [50]	Randomized double-blind placebo-controlled trial Duration: 4 months	38 women (50–65 y)	Menopause	Isoflavone tablets (80 mg/d; 60.8 mg genistein; 16 mg daidzein; 3.2 mg glycitein)	GDS	No significant effect
Singhal et al., 2016; India [51]	Comparative clinical Trial Duration: 3 months weeks	100 women (46–55 y)	Menopausal Vasomotor symptoms	Isoflavone tablets (60 mg/d)	ZSDS	Significant effect compared with control (*p* = 0.02)
Terauchi et al., 2014; Japan [53]	Randomized double-blind placebo-controlled pilot trial Duration: 8 weeks	91 women (40–60 y)	Menopausal symptoms	Grape seed Proanthocyanidin tablets (100 or 200 mg/d; 85% proanthocyanidins; 15% flavan-3-ols)	HADS	No significant effect
van Dongen et al., 2000; Netherlands [54]	Randomized double-blind placebo-controlled parallel group multicenter trial Duration: 24 weeks	123 subjects (mean age 82.8) (men and women)	Age-associated Memory impairment	*Ginko biloba* tablets (160 or 240 mg/d; 24% ginkgo flavonols)	GDS	No significant effect
Wahner-Roedler et al., 2011; US [55]	Randomized double-blind placebo-controlled early phase trial. Duration: 6 weeks	28 women (mean age 53.9 y)	Fibromyalgia symptoms	Soy shake (N/A mL/d; 160 mg soy isoflavones)	CES-D	No significant effect
Xiao et al., 2016; US [56]	Open-label Trial Duration: 8 weeks	10 subjects (18–70 y) (men and women)	Alcohol abuse and bipolar disorder	Icariin capsules (300 mg/d)	HAM-D	Significant effect compared with baseline (*p* = 0.01)
Zarghami et al., 2018; Iran [57]	Randomized double-blind clinical trial Duration: 6 weeks	25 subjects (18–70 y) (*n* = 19 women; *n* = 6 men)	Mild depression	*Asperugo procumbens* capsules (1.2 g/d; 6 mg total flavonoids)	HRSD	Significant lower effect compared with antidepressant control (*p* = 0.03)
Zhang et al., 2013; China [58]	Randomized double-blind placebo-controlled pilot trial Duration: 5 weeks	46 subjects (mean age: 25.7 y) (*n* = 23 women; *n* = 23 men)	Reward function in healthy subject	Green tea (400 mg/d; EGCG 45.6%; epigallocatechin 16.7%; epicatechin-3-gallate 11.4%; epicatechin 6.8%)	MADRS And HRSD	Significant effect compared with control (MADRS *p* < 0.01; HRSD *p* < 0.001)

GDS, Geriatric Depression Scale; HRSD, Hamilton Rating Scale for Depression; HAM-D, Hamilton Depression Rating; ZSDS, Zung Self-Rating Depression Scale; POMS, Profile of Mood States; QIDS-C, Quick Inventory of Depressive Symptomatology Clinician-Rated; HADS, Hospital Anxiety and Depression Scale; CES-D, Center of Epidemiologic Studies of Depression; EGCG, Epigallocatechin-gallate; BDI, Beck Depression Inventory; N/A, Not Available; GCS, Greene Climacteric Scale; MADRS, Montgomery–Asberg Depression Rating Scale; Peg–IFN, Pegylated Interferon.

**Table 2:** Data summary of observational studies assessing the effects of flavonoids on depressive symptoms.

Study (Author, Year, Country, Ref.)	Study Design	Subjects and Sample Size	Main Variable	Exposure Measure	Depression Scale	Results
Chang et al., 2016; US [66]	Longitudinal cohort study Duration: 1976–2001	82,648 women who participated in the NHS (mean age of 67 y at baseline) and NHSII (mean age of 47 y at baseline)	Dietary Flavonoid intake	FFQ (total flavonoid intake ranged from 127.6 to 779.4 mg/d)	CES-D and GDS	Participants in the highest flavonoid consumption group had a 7–10% reduction in depression risk compared with the lowest intake group (*p*-trend = 0.0004–0.08). Flavones and proanthocyanidins showed the strongest associations
Cui et al., 2020; Japan [74]	Cross-sectional study Duration: 2008–2011	1335 men (19–83 y)	Dietary Isoflavone intake	DHQ (isoflavone intake ranged from ≤10.61 to ≥25.79 mg/d)	SDS	A high level of dietary isoflavone intake was associated with a lower prevalence of depressive symptoms in all the adjusted models (*p*-trend = 0.002–0.029)
Godos et al., 2018; Italy [67]	Cross-sectional study Duration: 2014–2015	1572 participants who participated in the MHELAS (18–92 y) (*n* = 912 women; *n* = 660 men)	Dietary Polyphenol intake	FFQ (total flavonoid intake ranged from 157.0 to 543.7 mg/d)	CES-D	Higher dietary intake of flavanones and anthocyanins was inversely associated with depressive symptoms (*p*-trend = 0.001)
Hakim et al., 2016; Malaysia [65]	Longitudinal cohort study Duration: 18 months	400 elderly participants (≥60 y) (*n* = 231 women; *n* = 169 men)	Dietary Isoflavone intake	DHQ (mean intake: isoflavones 19.1 mg/d, daidzein 11.7 mg/d, genistein 7.6 mg/d)	GDS	No association between isoflavone intake and depression
Miyake et al., 2018; Japan [68]	Cross-sectional study	1745 pregnant women (mean age 31 y) who participated in the KOMCHS (an ongoing prospective prebirth cohort study)	Soy Isoflavone intake	DHQ (isoflavone intake ranged from 10.5 to 49.4 mg/d)	CES-D	Isoflavone intake was associated with a lower prevalence of depressive symptoms during pregnancy in all the quartiles (*p*-trend = 0.002)
Miyake et al., 2018; Japan [69]	Cross-sectional study	1744 pregnant women (mean age 31.2 y) who participated in the KOMCHS	Dietary patterns	DHQ (maximum isoflavone intake: Healthy pattern 43.5 mg/d Japanese pattern 32.7 mg/d Western pattern 31.2 mg/d)	CES-D	The healthy and Japanese patterns were inversely associated with depressive symptoms during pregnancy (Healthy pattern *p*-trend< 0.0001; Japanese pattern *p*-trend = 0.008)
Nagata et al., 1999; Japan [70]	Cross-sectional study Duration: September 1996– August 1997	284 menopausal women (mean age 47.1 y)	Soy product intake	FFQ (total isoflavone intake from soy product: 38.6 mg/d; isoflavone intake from fermented soy product 12.6 mg/d)	CES-D	No association between soy products and depression
Richard et al., 2014; Switzerland [71]	Cross-sectional study Duration: 2005–2008	193 perimenopausal women who participated in the NHANES (mean age 49 y)	Urinary phyto- estrogens	HPLC-APPI-MS/MS (mean value depressed vs. non-depressed women: isoflavones 128.3 and 97.4 μg/g; daidzein 57.4 and 48.3 μg/g; genistein 27.6 and 21.6 μg/g)	PHQ-9	No association between urinary isoflavone concentrations and depression
Rosli et al., 2019; Malaysia [72]	Cross-sectional study	349 participants with mild mental health problem (45–59 y) (*n* = 228 women; *n* = 121 men)	Dietary Polyphenols intake	FFQ (total flavonoid intake 265 mg/d)	GHQ-28	No association between flavonoid intake and depression
Woo et al., 2006; Hong Kong [73]	Cross-sectional study	3999 participants (≥65 y) (women and men)	Nutrient intake	FFQ (total isoflavone intake ranged from 4 to ≥19 mg/d)	GDS	No association between isoflavone intake and depression

DHQ, Diet History Questionnaire; GDS, Geriatric Depression Scale; NHS, Nurses’ Health Study; FFQ, Food-Frequency Questionnaire; SDS, Self-rating Depression Scale; MHELAS, Mediterranean Healthy Eating, Lifestyle and Aging Study; CES-D, Center of Epidemiologic Studies of Depression; SDS, KOMCHS, Kyushu Okinawa Maternal and Child Health Study; NHANES, National Health and Nutrition Survey; Patient Health Questionnaire-9; HPLC-APPI-MS/MS, High Performance Liquid Chromatography/Atmospheric Pressure Photoionization//Tandem mass spectrometry; GHQ-28, General Health Questionnaire-28.

## Supplementary Materials

The following are available online at https://www.mdpi.com/article/10.3390/antiox10111644/s1, Figure S1: Risk of bias assessment for the included randomized controlled trials, Table S1: Quality assessment of included observational studies using the New-Castle Ottawa Scale.

## Abbreviations

BDI: Beck Depression Inventory; CES-D, Center for Epidemiological Studies Depression Scale; CNS, central nervous system; DHQ, Diet History Questionnaire; FFQ, Food Frequency Questionnaire; GDS, Geriatric Depression Scale; HAM-D, Hamilton Depression Scale; HPA, hypothalamic–pituitary–adrenal; MDD, major depressive disorder; NOS, Newcastle–Ottawa Scale; PRISMA, Preferred Reporting Items for Systematic Reviews and Meta-Analysis; RCT, randomized controlled trial; WHO, World Health Organization.
