# Supplementary material for: Exploring the Impact of Flavonoids on Symptoms of Depression: A Systematic Review and Meta-Analysis
Source: Antioxidants (Basel). 2021 Oct 20;10(11):1644. doi: 10.3390/antiox10111644 (PMC8615051; doi:10.3390/antiox10111644)

Davinelli S. Exploring the Impact of Dietary Flavonoids on Symptoms of Depression: A Systematic Review and Meta-Analysis.

Supplementary Figure S1.

Risk of bias assessment for the included randomized controlled trials.

(a) Risk of bias graph

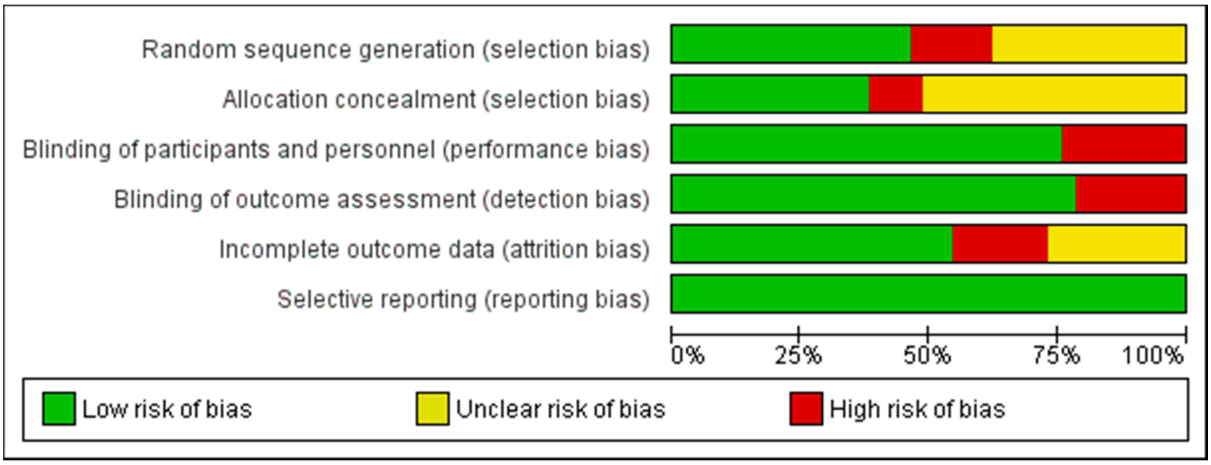

(b) Risk of bias summary. Based on the Cochrane Collaboration's tool for assessing risk of bias, + indicates low risk of bias, - indicates high risk of bias, and ? indicates unclear risk of bias.

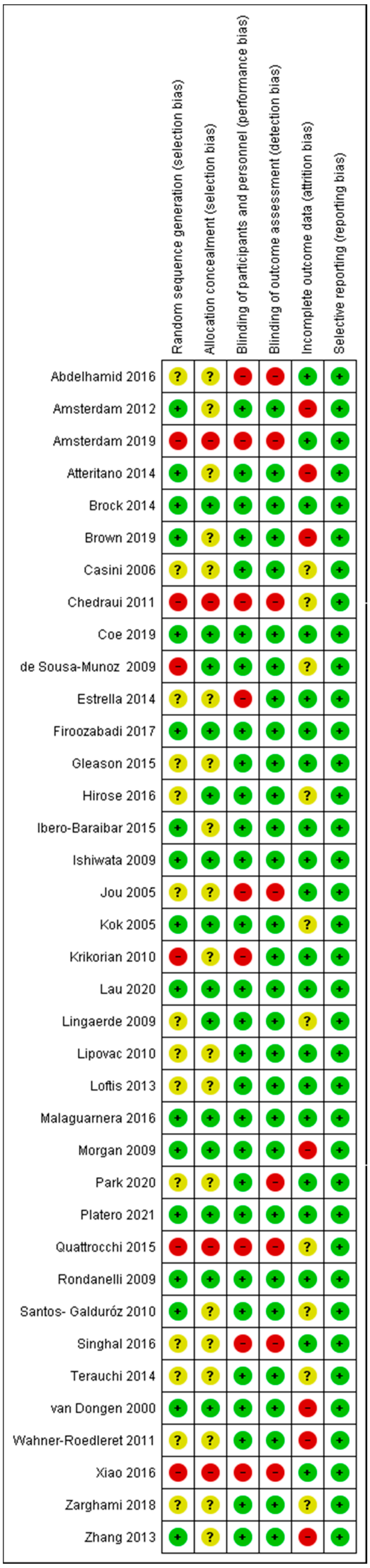

Supplement: Supplementary file 1 [file antioxidants-10-01644-s001.zip › Supplementary Figure S1.pdf]
