# Supplementary material for: Exploring the Impact of Flavonoids on Symptoms of Depression: A Systematic Review and Meta-Analysis
Source: Antioxidants (Basel). 2021 Oct 20;10(11):1644. doi: 10.3390/antiox10111644 (PMC8615051; doi:10.3390/antiox10111644)
Supplement: Supplementary file 1 [file antioxidants-10-01644-s001.zip › Supplementary Table S1.pdf]

Supplementary Table S1.

Quality assessment of included observational studies using the New-Castle Ottawa Scale

	SELECTION				COMPARABILITY	OUTCOME			
	Representativeness of the exposed cohort	Selection of the unexposed cohort	Ascertainment of exposure	Demonstration that the outcome of interest was not present at the start of the study	Comparability of cohorts on the basis of the design or analysis	Assessment of outcome	Was follow-up long enough for outcomes to occur?	Adequacy of follow-up of cohorts	Total
Hakim et al. 2016	*		*	*	**		*		6
Chang et al et al., 2016	*		*	*	**	*	*	*	8
Cui et al., 2020			*		**				3
Godos et al., 2018	*		*		**				4
Miyake et al., 2018	*		*		**				4
Miyake et al., 2018	*		*		**				4
Nagata et al., 1999	*		*		**				4
Richard et al., 2014	*		*		**				4
Rosli et al., 2019	*		*		**				4
Woo et al., 2006	*		*		**	*			5

[1]NOS high-quality choices are identified with a star. A maximum of 1 star is applied for each item within the Selection and Outcome categories, and a maximum of 2 stars for Comparability. NOS, Newcastle-Ottawa Scale; ref, reference.
